# In Vitro Effects of 0.05% Cetylpyridinium Chloride and 1% Povidone Iodine on Flexural Strength of Nickel-titanium Orthodontic Wires -

**DOI:** 10.31661/gmj.v14iSP1.3969

**Published:** 2025-12-15

**Authors:** Manijeh Mohammadian, Parisa Ghiasi, Milad Soleimani

**Affiliations:** ^1^ Department of Dental Biomaterials, School of Dentistry, Iran University of Medical Sciences, Tehran, Iran; ^2^ Student Research Committee, Alborz University of Medical Sciences, Karaj, Iran; ^3^ Department of Orthodontics, School of Dentistry, Shahid Beheshti University of Medical Sciences, Tehran, Iran

**Keywords:** Cetylpyridinium, Povidone-iodine, Mouthwashes, Orthodontic Wires, COVID-19

## Abstract

**Background:**

This study assessed the effects of 0.05% cetylpyridinium chloride (CPC) and 1% povidone iodine (PI) on flexural strength of nickel-titanium (NiTi) orthodontic wires.

**Materials and Methods:**

In this in vitro, experimental study, 27 pieces of NiTi orthodontic wires were randomly assigned to three groups (n=9) for immersion in 0.05% CPC, 1% PI, and distilled water (control) at 37°C for 90 minutes. After immersion, the modulus of elasticity, the yield strength, the mean force during the loading and unloading phases at 0.5 mm intervals of each wire (0.5, 1, 2.5, 2, and 2.5 mm), and the flexural strength of the wires were measured by the three-point bending test. Surface topography and corrosion of the wires were also inspected under a scanning electron microscope (SEM). Data were analyzed by one-way ANOVA and Tukey test (alpha=0.05).

**Results:**

CPC significantly increased the flexural strength of the wires (P0.05); while, the flexural strength was not significantly different in the PI and control groups (P0.05). CPC significantly increased the generated force during loading at all bending points and during unloading at 0.5- and 1-mm points (P0.05). PI increased the generated force during loading at 0.5 and 1 mm, and during unloading at 2.5 mm point (P0.05). CPC and PI had no significant effect on the yield strength in loading and unloading phases (P0.05). CPC and PI caused superficial corrosion of the wires.

**Conclusion:**

CPC (0.05%) and PI (1%) increased the mean force generated during unloading of the wires, their modulus of elasticity, and flexural strength.

## Introduction

Orthodontic treatment typically involves the use of metallic wires and brackets crafted from various metal alloys [1]. Yet, these metal elements are subject to degradation in the oral cavity due to chemical, mechanical, thermal, microbial, and enzymatic changes, leading to the release of ions [2]. Such ion release may result in staining of nearby soft tissues, allergic responses, or localized discomfort. Furthermore, when ion concentrations reach specific thresholds, they may pose toxic biological risks [3][4]. Evidence shows higher ion release from NiTi than stainless-steel alloys [5][6]. Orthodontic wires should be able to generate light continuous forces [7][8][9] capable of triggering a biological response in the periodontal ligament for physiological bone remodeling [9][10] with minimal patient discomfort and tissue damage such as hyalinization and root resorption, and within the fastest time possible [11][12]. Also, orthodontic wires must be able to withstand mechanical, thermal, and chemical tensions in the oral environment [13]. Currently, NiTi wires are increasingly used due to their optimal flexibility [7][10][14], spring-back [7][10][13][14], generating light continuous forces [7][9][10][13][14], shape memory [7], friction resistance [7], and biocompatibility [7] in the first phase of orthodontic treatment [14].

Nickel is used in the composition of orthodontic wires since it confers shape memory and super-elasticity [15]. Nonetheless, nickel-containing alloys undergo corrosion and release nickel ions, which can lead to allergic reactions. The magnitude of nickel release varies depending on the immersing solution [16]. Titanium is also used in the composition of archwires to confer flexibility and increase corrosion resistance [17]. Release of titanium from archwires is insignificant and too low to be measurable (<30 ppb) [6].

Fixed orthodontic appliances generally interfere with oral hygiene practice [8][14][18], and complicate mechanical microbial plaque removal by toothbrushing [19][20]. Due to the electrostatic reactions, bacteria tend to adhere to metal surfaces [18] and lead to plaque accumulation around the bracket base [8][14][18]. They cause enamel demineralization and smooth-surface caries during orthodontic treatment [8][14][18][21], and create an imbalance between the demineralization and remineralization cycles [18], resulting in caries and acute gingival inflammation [18][19].

Different protocols such as antimicrobial agents are used for reduction of bacterial plaque and risk of periodontal disease during the active phase of orthodontic treatment [19]. Cetylpyridinium chloride (CPC) is a water-soluble quaternary ammonium compound that has been extensively studied for its potent antimicrobial activity against a wide range of microorganisms, including bacteria, viruses, and fungi [22][23][24][25][26][27]. CPC is commonly incorporated into oral care products, including mouthwashes, toothpastes, and orthodontic adhesives, as well as in food safety and biomedical applications, demonstrating versatility in both clinical and industrial settings [22]. Povidone iodine (PI) is a stable chemical complex of polyvinyl pyrrolidone and iodine, which is a broad-spectrum antimicrobial agent used for infection control [28][29][30][31].

Mouthwashes are routinely prescribed by orthodontists to prevent caries and gingival inflammation during the course of orthodontic treatment [32]. Thus, orthodontic wires are continuously exposed to mouthwashes, which may alter their mechanical properties [33][34]. In addition to fluoride solutions, CPC and PI are often recommended as adjunctive agents in orthodontic patients to minimize microbial accumulation. However, little is known about their potential impact on the mechanical performance of NiTi wires. Considering the significance of flexural strength of archwires for orthodontic tooth movement [13], this study aimed to assess the effects of 0.05% CPC and 1% PI on the flexural strength of NiTi orthodontic wires. The null hypothesis was that CPC and PI would not cause significant changes in the flexural strength of NiTi wires.

## Materials and Methods

This in vitro, experimental study was conducted on 27 pieces of NiTi orthodontic wires with a round cross-section. The study protocol was approved by the ethics committee of Alborz University of Medical Sciences (IR.ABZUMS.REC.1400.279).

### Sample Size

The required sample size was determined to be at least 3 per group, based on Aghili et al.'s study [35], utilizing the power analysis tool in PASS 11, with parameters set at α=0.05, β=0.2, a standard deviation of 0.19, and an effect size of 1.47.

### Specimen Preparation

A total of 27 pieces were cut with 4 cm length from the straight end of 0.016-inch round NiTi wires (American Orthodontics, USA). After cutting, the pieces were measured to ensure their equal length, and those shorter or longer than 4 cm were excluded and replaced. Eligible pieces were randomly assigned to 3 groups (n=9) of distilled water (control), 0.05% CPC (Iran Najo Co, Terhan, Iran), and 1% PI (Walgreens, Illinois, USA) using the Rand feature of Excel software.

**Figure-1:**
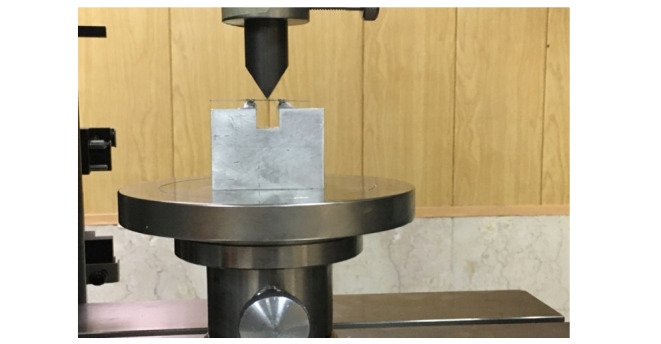
Figure 1. Loading of a wire in the three-point bending test

The wire pieces in each group were immersed in the respective solutions in non-reactive 10 mL plastic tubes and incubated at 37°C for 90 minutes. Next, they were rinsed with saline and transferred into new coded plastic tubes. Subsequently, they were subjected to the three-point bending test in a universal testing machine (Z050 Zwick/Roell; Zwick GmbH & Co.KG., Ulm, Germany). An aluminum jig measuring 3 x 4 x 5 cm was used for this test which had a notch with 1 cm width and 1 cm depth on one of its surfaces. Two orthodontic brackets were glued to the borders at the two sides of this notch with 15.5 mm distance from each other according to the Wilkinson standards and fixed with cyanoacrylate glue. The wires were inserted into the brackets and secured using elastomeric O-rings (Figure-1).

Compressive load was applied by a rod vertically attached to the machine. The load was applied to the midpoint of each wire at a crosshead speed of 0.5 mm/minute. Each wire was loaded until experiencing 3 mm of displacement, and was then returned to its baseline state by the same speed. The load in Newtons (N) and the displacement in millimeters (mm) were recorded separately for each specimen using the testXpert R software (Zwick GmbH & Co. KG). The mean force was calculated at 0.5 mm bending intervals of each wire (0.5, 1, 1.5, 2, and 2.5 mm) during the loading and unloading phases. According to the load-displacement curve and wire dimensions, the modulus of elasticity was calculated using the following formula [36]:


E\,(\mathrm{GPa}) = \frac{m L^{3}}{12 \pi R^{4}} \times 0.001

Where E is the modulus of elasticity in gigapascals (GPa), L is the wire support length in millimeters (mm), R is the wire radius in millimeters (mm), and m is the gradient of the linear part of the curve during the loading and unloading phases (N/mm).

The yield strength was calculated using the following formula [37]:


YS = \frac{F L}{\pi R^{3}}

Where YS is the yield strength in megapascals (MPa), F is the force at the point of yield strength at the end of the straight line in Newtons (N), L is the wire support length in millimeters (mm), and R is the wire radius in millimeters (mm).

The following formula was used to calculate the flexural strength [37]:


\delta_{fs} = \frac{F_{\max} L}{\pi R^{3}}

Where δfs is the final flexural strength, F is the maximum force at the end of the curve in Newtons (N), L is the wire support length in millimeters (mm), and R is the wire radius in millimeters (mm). Three wires were randomly selected from each group, gold sputter coated with 200 nm thickness, and the surface topography and corrosion of the wires were assessed under a scanning electron microscope (SEM; XL30, Philips Holand) at x1000 and x5000 magnifications.

### Statistical Analysis

Normal distribution of data was confirmed by the Shapiro-Wilk test (P>0.05). Accordingly, comparisons were made by one-way ANOVA and Tukey test. All statistical analyses were carried out using SPSS version 24 at 0.05 level of significance.

## Results

**Figure-2:**
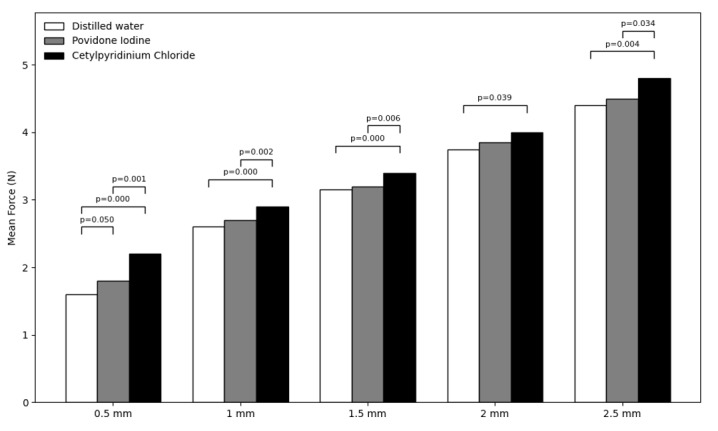
Figure 2. Mean force at different intervals during the loading phase in the three groups

**Figure-3:**
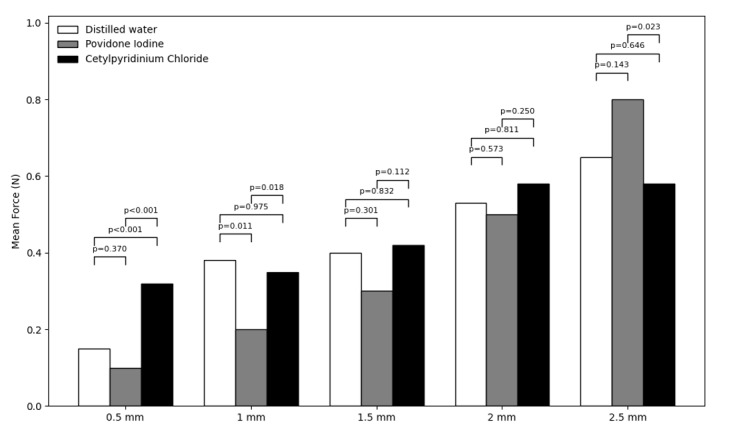
Figure 3. Mean force at different intervals during the unloading phase in the three groups

**Figure-4:**
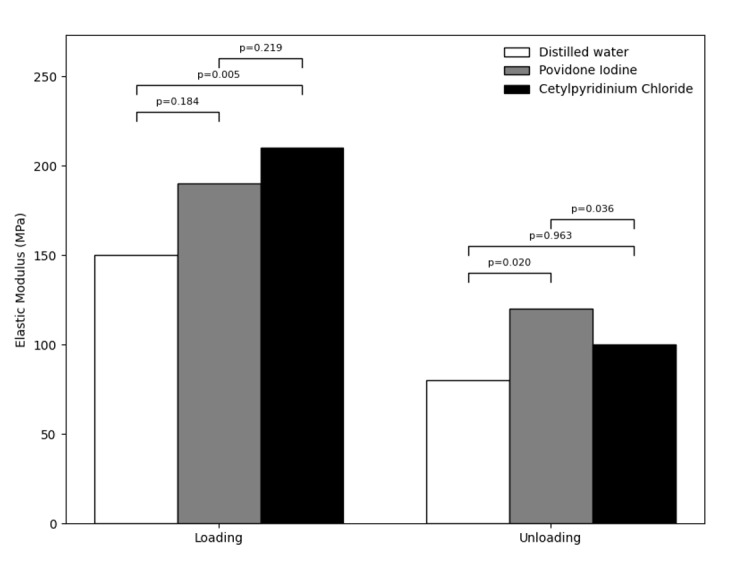
Figure 4. Modulus of elasticity of the NiTi wire in the three groups during the loading and unloading phases

**Figure-5:**
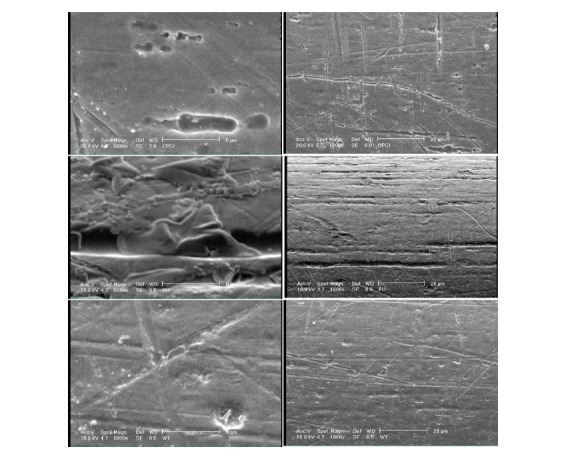
Figure 5. SEM micrographs of the surface of NiTi wires at x1000 and x5000 magnifications following immersion in CPC (A), PI (B) and distilled water (C)

**Table:** Table1. Measures of Central Dispersion for the Flexural Strength of NiTi Wires in the Three Groups (n=9)

Group	Mean	Std. Deviation	95% Confidence Interval for Mean		Minimum	Maximum
			Lower Bound	Upper Bound		
0.05% CPC	3131.31	163.29	3005.79	3256.83	2948.17	3430.99
1% PI	2884.33	279.27	2669.66	3099.00	2588.90	3483.94
Distilled water	2827.84	130.43	2727.58	2928.10	2662.92	3024.46

**Table:** Table2. Measures of Central Dispersion for the Yield Strength (MPa) of the NiTi Wire in the Three Groups During the Loading and Unloading Phases (n=9)

**Phase**	**Group**	**Mean**	**Std. deviation **	**95% confidence interval **		**Minimum**	**Maximum**
				**Lower bound **	**Upper bound **		
	CPC	1133.98	70.34	1079.91	1188.05	1014.04	1267.97
Loading	PI	1202.54	88.27	1134.68	1270.39	1123.97	1298.87
	Distilled water	1171.81	74.36	1114.65	1228.97	1100.01	1341.42
	CPC	797.23	315.36	554.82	1039.65	458.34	1484.90
Unloading	PI	955.83	189.65	810.05	1101.62	621.18	1151.82
	Distilled water	867.83	173.55	734.43	1001.24	724.80	1294.17

### Flexural Strength

Table-1 presents the measures of central dispersion for the flexural strength of the NiTi wires in the three groups. As shown, the CPC group showed the highest, and the distilled water group showed the lowest flexural strength. The difference in flexural strength was significant among the three groups. Pairwise comparisons by the Tukey’s test showed that the mean flexural strength of the CPC group was significantly higher than that of the PI group (P=0.04) and distilled water (P=0.01) group; however, the difference in flexural strength between the PI and distilled water groups was not significant (P=0.0824).

### Force

Figure-2 shows the mean force at different bending intervals during the loading phase in the three groups. As shown, the highest mean force was noted at 2.5 mm bending point in the CPC group, and the lowest mean force was recorded at 0.5 mm bending point in the distilled water group. Comparison of the three groups regarding the force at different intervals during the loading phase revealed significant differences among the three groups at 0.5 mm (P<0.01), 1 mm (P<0.01), 1.5 mm (P<0.01), 2 mm (P=0.046), and 2.5 mm (P=0.004) intervals. Pairwise comparisons showed the greatest difference in the mean force between the CPC and distilled water groups at 0.5 mm bending point, and the minimum difference between the PI and distilled water groups at 1.5 mm bending point. The distilled water group had significant differences with the CPC group at all intervals (P<0.05), but had no significant difference with the PI group (P>0.05). Also, the difference between the CPC and PI groups was significant at all intervals (P<0.05) except at 1.5- and 2-mm points (P>0.05). Figure-3 shows the mean force at different intervals during the unloading phase in the three groups. As shown, the highest mean force was noted at 2.5 mm bending point in the PI group, and the lowest mean force was recorded at 1.5 mm bending point in the distilled water group. Comparison of the three groups regarding the force at different intervals during the unloading phase revealed significant differences among the three groups at 0.5 mm (P<0.01), 1 mm (P=0.006), and 2.5 mm (P=0.026) points. Pairwise comparisons showed the greatest difference in the mean force between the CPC and PI groups at 0.5 mm point, and the smallest difference between the CPC and distilled water groups at 1 mm point. The difference between the distilled water and CPC groups was not significant at any point (P>0.05) expect at 0.5 mm point (P<0.05). The difference between the distilled water and PI groups was not significant at any point (P>0.05) except at 1 mm (P<0.05). However, the difference between the CPC and PI was significant at all points (P<0.05) except at 1.5- and 2-mm points (P>0.05).

### Modulus of Elasticity

Figure-4 shows the modulus of elasticity of the wire in the three groups during the loading and unloading phases. As shown, the highest modulus of elasticity during the loading phase was recorded in the CPC group (220.81 GPa); while, the lowest was recorded in the distilled water group (155.39 GPa). During the unloading phase, the highest modulus of elasticity was recorded in the PI group (125.03 GPa) and the lowest in the distilled water group (87.38 GPa). A significant difference was noted in the modulus of elasticity of the NiTi wire in the three groups during both the unloading (P=0.013) and the loading phase.

Pairwise comparisons showed the largest difference between the CPC and distilled water groups during the loading phase, and the smallest difference between these two groups during the unloading phase. The mean modulus of elasticity of the wires in the PI group had significant differences with the CPC and distilled water groups during the unloading phase (P<0.05). No other significant differences were found (P>0.05).

### Yield Strength

Table-2 shows the yield strength of the NiTi wire in the three groups during the loading and unloading phases. As shown, the highest mean yield strength was recorded in the PI group in the loading phase and the lowest in the CPC group in the unloading phase. The greatest difference was found between the CPC and PI groups in the unloading phase, and the smallest difference between the PI and distilled water groups in the loading phase. Nonetheless, the three groups had no significant difference in the yield strength neither in the loading nor in the unloading phase (P>0.05).

### SEM

Figure-5 shows SEM micrographs of the surface of NiTi wires at x1000 and x5000 magnifications in the three groups. As shown, the surface was damaged in all three groups. NiTi wires exposed to PI showed larger surface defects in the form of pits, deeper grooves, and white corrosion products in the pits, compared with the CPC group. The wires in the CPC group showed a smoother surface. Wire surface destruction in the CPC group was insignificant compared with the distilled water group.

## Discussion

This study assessed the effects of 0.05% CPC and 1% PI on flexural strength of NiTi orthodontic wires. The present results showed that the mean flexural strength of the CPC group was significantly higher than that of the PI and distilled water groups; however, the difference in flexural strength between the PI and distilled water groups was not significant. Thus, the null hypothesis of the study was rejected. Also, the 0.05% CPC and 1% PI had significant effects on the mean force at 0.5-, 1-, 1.5-, 2-, and 2.5-mm intervals during the loading, and 0.5-, 1-, and 2.5-mm intervals during the unloading phase.

Nickel and titanium ions are released following exposure of uncoated NiTi wires to the saliva. Following formation of a titanium oxide protective layer, wire corrosion and titanium ion release decrease; however, exposure to fresh saliva with a low pH may lead to dissolution of the titanium oxide protective layer, and subsequently result in advanced corrosion, causing the release of nickel ions, although insignificant [38]. The amount of released nickel ions is often too low to cause allergic reactions [39][40]. Nonetheless, they can accumulate in the gingiva and cause hyperplasia [41]. Repeated corrosions can affect wire flexibility and the magnitude of load under loading and unloading conditions, causing a reduction in flexural properties of uncoated NiTi wires [42].

The mean force of NiTi wire was the highest in the CPC group at all intervals during the loading phase (the required force for wire engagement in the bracket). During the unloading phase (force applied by the wire to the teeth during the course of orthodontic treatment), the mean wire force was the highest in the CPC group at 0.5 and 1 mm, and in the PI group at 2 mm bending point. During the loading phase, CPC increased the force in NiTi wires at all points while PI increased the force at 0.5- and 2-mm intervals compared to the distilled water group. During the unloading phase, CPC increased force at 0.5 and 1 mm, and PI increased force at 2.5 mm point compared with the distilled water group. According to the present results, it appears that 0.05% CPC and 1% PI increase the loading and unloading forces; this effect may be due to the protective role of the titanium oxide layer, which is formed within a short period of time and prevents wire surface corrosion. Subsequently, the unloading forces serve as a reciprocating force and when the orthodontic wire returns to its original shape, it affects the periodontal ligament and tooth movement biomechanics [43]. It appears that in treatment with NiTi wires, use of CPC and PI mouthwashes can improve clinical performance. Similarly, Mlinaric et al. [44] compared the effects of hyaluronic-acid based edible disinfectants and chlorhexidine on NiTi wires and reported that they did not damage the titanium oxide protective layer and had no adverse effect on the properties of NiTi wires. The pH of mouthwashes is another important parameter, which can affect the corrosion resistance of wires. A more acidic pH can cause a greater reduction in mechanical force of orthodontic wires [14]. More acidic solutions with a lower pH further penetrate into the protective coating of NiTi wires and increase wire corrosion. CPC has a pH of around 6.5 [45]. Unlike the present results, Alavi et al. [14] showed that daily use of a fluoride mouthwash with a higher acidity (pH of 4) caused greater reduction of mechanical properties of NiTi wires in the unloading phase. Difference between their results and the present findings can be due to using a different solution with a different concentration and a more acidic pH in their study, compared with the present investigation.

To the best of the authors’ knowledge, no previous study has assessed the effects of CPC and PI mouthwashes on orthodontic wires to compare our results with. However, similar results have been reported in using other mouthwashes. For instance, Katic et al. [46] reported insignificant release of nickel from rhodium-coated NiTi wires following their immersion in prophylactic agents with high fluoride content, causing an improvement in mechanical properties of NiTi wires. Unlike the present study, Hashim and Al-Joubori [47] showed that the concentration of fluoride had a significant effect on the NiTi wire force, and the unloading forces significantly decreased in the wires subjected to neutral fluoride, compared with stannous fluoride gel or Phos-Flur mouthwash. Such a different result may be due to using a different type of mouthwash and shorter immersion time.

In the current study, the flexural strength was significantly higher in wires immersed in 0.05% CPC while the difference in this regard was not significant between the PI and distilled water groups. Similarly, Kupka et al. [48] showed a significant increase in hardness and flexural strength of glass ionomers that contained 0.5% to 2% CPC in their composition, compared with the control specimens. They explained that CPC is a quaternary ammonium salt capable of interaction with poly acrylic acid [45].

Stiffer wires have a higher modulus of elasticity [49]. In the present study, 0.05% CPC and 1% PI increased the modulus of elasticity of NiTi wires during the loading phase compared with distilled water. During the unloading phase, 1% PI yielded the highest modulus of elasticity. Thus, CPC and PI increased the stiffness and corrosion resistance of the NiTi wire. This result was expected considering the increase in force and flexural strength of wire in the CPC and PI groups. Unlike the present results, Mlinaric et al. [44] showed insignificant effect of hyaluronic-acid based disinfectants, chlorhexidine, and Listerine on the modulus of elasticity of uncoated NiTi wires, and those with nitride and rhodium coatings. This difference can be due to their coatings and different types of mouthwashes used, which is more important than the type of coating [44].

Consistent with the present results, Aghili et al. [35] reported an increase in the modulus of elasticity of NiTi wires due to the effect of sodium fluoride, chlorhexidine, and Zataria extract. However, Hammad et al. [8] reported that application of 1.1% acidulated phosphate fluoride gel decreased the modulus of elasticity of composite-reinforced wires, which can be due to high acidity of the fluoride compound and resultantly damaged glass fillers of the composite, and surface corrosion.

The yield strength is the maximum stress a wire can tolerate before undergoing plastic deformation. The results showed that the yield strength of the wire was not influenced by the tested mouthwashes, which is an advantage. Unlike the present results, Mane et al. [50] reported a significant reduction in yield strength in the unloading phase following exposure of NiTi and copper-NiTi wires with a rectangular cross-section to acidulated phosphate fluoride. This result can be due to using fluoride gel and release of hydrogen ions [50].

However, Gupta et al. [51] found no significant difference in the yield strength of NiTi wires in the loading phase following exposure to Phos-Flur and Prevident 5000 gel; however, significant changes occurred in the yield strength during the unloading phase, which can be due to greater corrosion of the wire as a result of high acidity of the solutions. Consistent with the present results, Sander [52] found no significant change in the yield strength during the loading and unloading phases of coated wires following exposure to neutral sodium fluoride gel, neutral sodium fluoride mouthwash, and acidulated phosphate fluoride, which was explained to be due to the polymer and rhodium coating of the wires. Variations in the reported results in the literature can be due to differences in the type and pH of the solutions/mouthwashes, their concentrations, and coating of the wires.

SEM assessment of the surface of NiTi wires exposed to CPC and PI revealed greater changes, higher number of pits and fissures, and greater amounts of corrosion products on the surface of wires in the PI group compared with the CPC group. However, these changes on the surface of wires in the CPC group were not significant compared with the distilled water group. Thus, the SEM findings were in accordance with the obtained results regarding the mechanical properties of NiTi wires exposed to the mouthwashes [42]. Similarly, previous studies reported greater corrosion and a reduction in mechanical properties of orthodontic wires following exposure to fluoride gel and mouthwash [51][53].

Although novelty was the main strength of the present study, lack of similar studies for the purpose of composition was a limitation. Also, this study had an in vitro design. Many factors can influence the results in the oral environment, such as the masticatory function, which were not taken into account in this study. Future studies are required to better simulate the clinical setting to obtain more generalizable results. Finally, the results should be verified in the clinical setting to cast a final judgment in this regard.

## Conclusion

CPC (0.05%) and PI (1%) mouthwashes increased the mean force generated during the unloading phase of NiTi wires, their modulus of elasticity, and flexural strength, and improved their mechanical properties. They also caused superficial corrosion of the wires.

## Conflict of Interest

The author declares that they have no competing interests.
